# ANCA positive relapsing polychondritis, Graves disease, and suspected moyamoya disease: A case report

**DOI:** 10.1097/MD.0000000000009378

**Published:** 2017-12-22

**Authors:** Yi-Yi Xuan, Tian-Fang Li, Lei Zhang, Sheng-Yun Liu

**Affiliations:** Department of Rheumatology and Immunology, First Affiliated Hospital of Zhengzhou University, Zhengzhou City, Henan Province, P.R. China.

**Keywords:** Graves disease, moyamoya disease, MPO-ANCA, propylthiouracil, relapsing polychondritis

## Abstract

**Ratioinale:**

Relapsing polychondritis (RP) is a rare and heterogeneous disease complex of unknown origin which basically affects cartilaginous structures, 40% of which accompanied by rheumatic, hematologic, and endocrine disease. Among them, vasculitis is the most common accompanying type and usually presented with positive antineutrophilic cytoplasmic antibody (ANCA). The presence of ANCA could be primary or drug-induced like propylthiouracil (PTU). Central involvement of RP is very rare, and there is almost no report of cerebral vasculopathy manifested as moyamoya.

**Patient concerns:**

A 26-year-old woman complained about recurrent fever, auricular chondritis, ocular inflammation, and arthritis. She had an 8-year drug intake of PTU for Graves disease. Myeloperoxidase antineutrophilc cytoplasmic antibodies (MPO-ANCA) were found positive. Magnetic resonance angiography (MRA) detected multiple intracranial vasculopathy which we highly suspected it as moyamoya disease.

**Diagnoses:**

Relapsing polychondritis, Graves disease and suspected moyamoya disease were clinically diagnosed.

**Interventions and outcomes:**

In case of possible PTU-induced vasculitis and the aggravation of vasculopathy, PTU was replaced by Iodine-131 (I^131^) therapy. Induction treatment included oral prednisone 30 mg daily and oral cyclophosphamide 100 mg daily. Symptoms rapidly relieved before discharge. Inflammation markers were normal and MPO-ANCA decreased in 3 weeks after admission. Prednisone was gradually tapered to 7.5 mg daily and at month 10 azathioprine was continued for maintenance.

**Lessons:**

RP can overlap with Graves disease and moyamoya disease; comprehensive tests should be performed when admission. When relapsing polychondritis is accompanied with Graves disease, especially when ANCA is positive, PTU should be avoided.

## 1 Introduction

Relapsing polychondritis (RP) is a rare disease classically characterized by recurrent inflammation of cartilaginous and proteoglycan-rich structures, which can basically affect all organs. One-third are accompanied by other rheumatic disease (common), hematologic disease, and endocrine disease (rare).^[1]^ Central involvement of RP is very rare and commonly with severe symptoms,^[1]^ whereas asymptomatic multiple intracranial vasculopathy manifested as moyamoya (which may later develop cerebralvascular event) has not yet been reported. Furthermore, moyamoya disease and Graves disease with RP simultaneously has barely been reported either.

Here, we report a case of RP who had a regular medication intake of propylthiouracil (PTU) for Graves disease for almost 8 years. After comprehensive examinations, we also found the presence of myeloperoxidase antineutrophilc cytoplasmic antibodies (MPO-ANCA) and cerebral involvement of asymptomatic multiple intracranial vasculopathy which we highly suspected it as moyamoya disease. As far as we know, it is an exceptionally rare occurrence of moyamoya disease and Graves disease presented in RP.

## 2 Case report

A 26-year-old woman was healthy until in October 2015, when both of her eyes suddenly became red, swelling, and painful. She complained about several episodes of redness and pain of auricles and eyes bilaterally for 9 months. The patient also suffered from fever and intermittent arthritis in both large and small joints including knee, wrist, elbow, hip, and metacarpophalageal joints. One week before hospitalization, she experienced another episode of pain, swelling, and redness of the left auricle. There was a past history of hyperthyroidism treated with PTU for 8 years.

Physical examinations showed the injected sclera; the red, swelling, and painful ears, both in normal shape; the swelling and tender ankles. The neurologic examination was negative.

**Figure 1:**
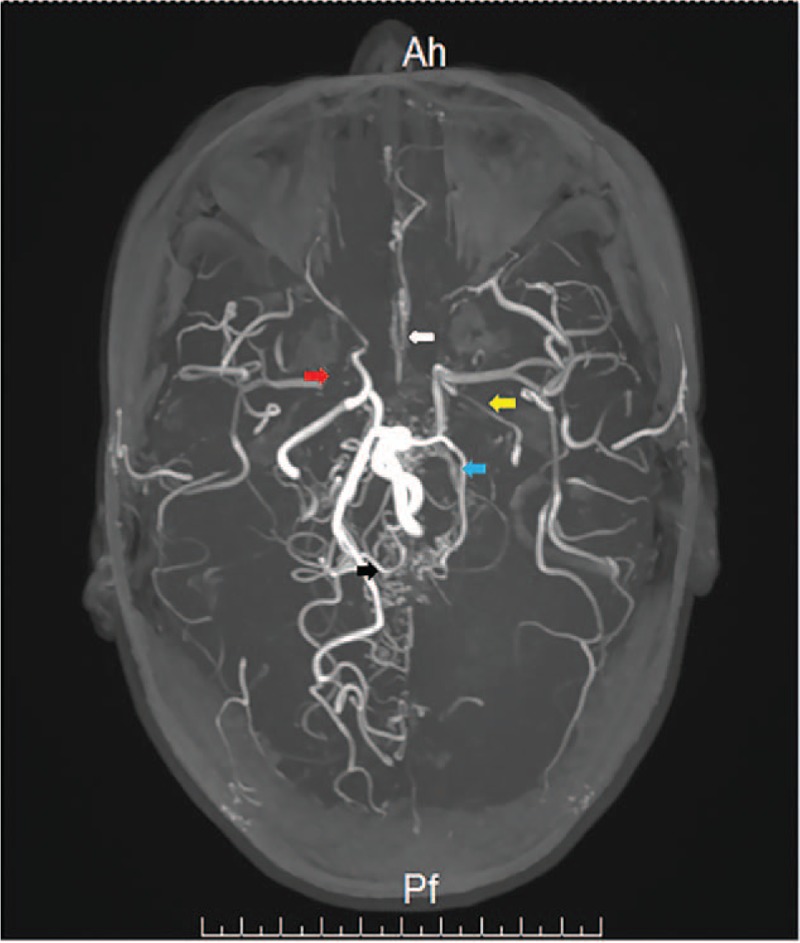
MRA examination showed that the intracranial segment of the left internal carotid artery (yellow arrow) and the distal segment of the right distal carotid artery were markedly thin. In addition, A1 segment of bilateral cerebral anterior (white arrow) was not found, and A2 segment was markedly thin. A near-complete occlusion was found in the M1 segment of the right middle cerebral artery (red arrow) with sparse distal branches. The left posterior cerebral artery (blue arrow) was also thin, with sparse distal branches. Compensatory multiple small “puffy smoke like” vessels were found in the saddle area, the Cisterna ambiens, and the parietal lobe near the middle line (black arrow).

Laboratory tests showed a raised level of erythrocyte sedimentation rate (ESR, 76 (0–20) mm/h) and C-reactive protein (CRP, 81.7 (0–10) mg/L). Immunological tests showed the presence of perinuclear-ANCA and MPO were 312.5 (0–20) RU/mL, although HLA-B27, anti-ENA antibodies (ENA, extractable nuclear antigen), and antinuclear antibodies (ANA) were negative. Serum levels of free triiodothyronine (FT3), free thyroxine (FT4), and thyroid-stimulating hormone (TSH) were 5.56 (3.28–6.47) pmol/L, 16.75 (7.9–18.4) pmol/L, and 0.01 (0.34–5.6) uIU/mL, respectively. Thyroid hormone antibody (TRAB) was 5.16 (0–1.75) IU/L. The complete metabolic panel and thorax CT scan were normal. However, the CT revealed multiple ischemic foci in the deep white matter in the left frontal lobe. Therefore, brain magnetic resonance imaging (MRI) and magnetic resonance angiography (MRA) were further performed to exclude any cerebrovascular disorders. The result showed multiple abnormalities of large blood vessels (Fig. 1).

We diagnosed ANCA positive relapsing polychondritis, suspected moyamoya disease and Graves disease. The initial treatment was prednisone (30 mg/d) and oral CTX (100 mg/d). PTU was withdrawn and Iodine-131 (I^131^) therapy was applied. At months 10, CTX was stopped and azathioprine (AZA, 50 mg/d, adjusted according to liver enzyme and white blood cell nadir) was continued for maintenance; prednisone was gradually tapered to 7.5 mg/d. At months 12, the MPO-ANCA was reexamined and decreased to 197.5 (0–20) RU/mL (Table 1). All symptoms were alleviated and inflammation markers returned to normal in 3 weeks after admission. No adverse events were observed. No flare has been found. Patient informed consent was given, and ethic committee approval is not necessary for this does not involve any animal or human trials.

**Table 1:**
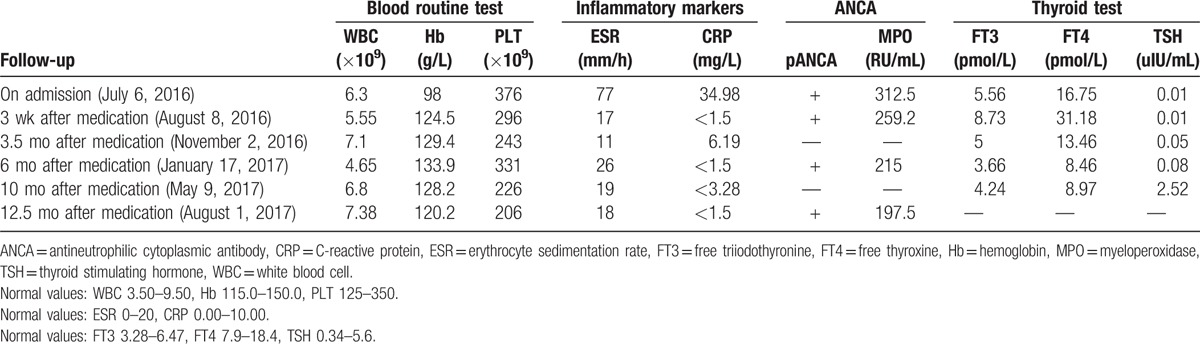
Important clinical indexes changes throughout the disease course.

## 3 Discussion

Relapsing polychondritis, Graves disease, and suspected moyamoya disease was clinically diagnosed. First, for the cerebral vasculopathy part, the diagnosis of moyamoya disease was jointly made by a MRI physician and a neurologist after thorough discussion since the patient refused the further examination of Digital Subtraction Angiography or Computed tomography angiography. And artery biopsy was not performed concerning that it was invasive and unnecessary, so the pathologic result was uncertain. Second, the diagnosis of relapsing polychondritis was definitive according to McAdam suggested criteria.^[2]^

RP is a complex disease entity that has numerous associations with autoimmune diseases, including vasculitis, systemic lupus erythematosus, rheumatoid arthritis, and also Graves disease.^[1]^ Although the pathophysiology of RPC still remains uncovered, Foidart et al^[3]^ found circulating antibodies against collagen components (type II, IX, XI) which may consequently cause inflammation and destruction of vessels and cartilages that is rich in collagen. Moreover, HLA-DR4 alleles also play a genetic role in RP which has some similarities to other rheumatic diseases.^[4,5]^ These may partly explain the overlap of the RP patient with the occurrence vasculopathy and Graves disease.

Intracranial involvement in RP is rare and severe with poor prognosis. Furthermore, cerebral involvement is mostly presumed to be caused by vasculitis^[6]^ which are often presented with positive ANCA and have symptoms like dyskinesia, sensory disability, headache, memory loss, stroke, and so forth.^[1]^ The patient only presented with imaging abnormalities for the moyamoya disease and deep white matter foci, but is still potentially risky. Kuroda et al^[7]^ reported that 20% asymptomatic moyamoya had cerebral infarction and 40% of them had aberrant cerebral hemodynamics, suggesting asymptomatic moyamoya disease may easily cause ischemic or hemorrhagic stroke. On the contrary, Balavoine^[8]^ reported the high prevalence of positive ANCA caused by PTU for Graves disease which varied between 4% and 64% with PTU (median 30%), and young age and the duration of drug intake were the main factors contributing to the emergence of ANCA positivity. Our patient has positive ANCA and an 8-year history of PTU intake which could induce ANCA and probably cause PTU-induced cerebral vasculitis.^[9]^ Balavoine^[8]^ also stated most of them were asymptomatic, but still 15% of them exhibited clinical evidence of vasculitis. From these 2 perspectives, 2 causes might account for the moyamoya disease: one was the vasculitis itself, and the other was the drug, PTU. Or the least possible idiopathic. Consequently, to avoid the deterioration of intracranial vasculopathy, I^131^ therapy was instead applied and the relatively aggressive initial treatment was adopted.

Some limitations blurred the connections for the overlapping of positive ANCA, relapsing polychondritis, PTU, and moyamoya disease. First, the biopsy result of the intracranial arteries was not obtained so that the pathology was unknown for whether it was ANCA associated or idiopathic. Second, there was no histology to support the RP although the clinical manifestation was classic. Third, the presence of ANCA was not always pathogenic in PTU-treated patients, with only 15% of which has clinical vasculitis. And in our case, the MPO-ANCA did not rapidly improve but slowly decreased, which did not accord with the rapid improvement of ANCA after the withdrawal of PTU in some published literature.

In conclusion, RP is still a complicated disease complex that can be coexisted with moyamoya disease and Graves disease. When RP is accompanied with Graves disease, especially when ANCA is positive, PTU should be avoided.
